# Psychometric Properties of a Generic, Patient-Centred Palliative Care Outcome Measure of Symptom Burden for People with Progressive Long Term Neurological Conditions

**DOI:** 10.1371/journal.pone.0165379

**Published:** 2016-10-25

**Authors:** Wei Gao, Vincent Crosby, Andrew Wilcock, Rachael Burman, Eli Silber, Nilay Hepgul, K Ray Chaudhuri, Irene J. Higginson, on behalf of the OPTCARE Neuro trial

**Affiliations:** 1 King's College London, Faculty of Life Sciences and Medicine, Department of Palliative Care, Policy and Rehabilitation, Cicely Saunders Institute, London, United Kingdom; 2 Palliative Medicine, Nottingham University Hospitals NHS Trust, Nottingham, United Kingdom; 3 Palliative Medicine and Medical Oncology, School of Medicine, University of Nottingham, Nottingham, United Kingdom; 4 Department of Neurology, Kings' College Hospital, London, United Kingdom; 5 National Parkinson Foundation Centre of Excellence, Kings College Hospital and Kings College, London, United Kingdom; Universidade Federal do Rio de Janeiro, BRAZIL

## Abstract

**Background:**

There is no standard palliative care outcome measure for people with progressive long term neurological conditions (LTNC). This study aims to determine the psychometric properties of a new 8-item palliative care outcome scale of symptom burden (IPOS Neuro-S8) in this population.

**Data and Methods:**

Data were merged from a Phase II palliative care intervention study in multiple sclerosis (MS) and a longitudinal observational study in idiopathic Parkinson’s disease (IPD), multiple system atrophy (MSA) and progressive supranuclear palsy (PSP). The IPOS Neuro-S8 was assessed for its data quality, score distribution, ceiling and floor effects, reliability, factor structure, convergent and discriminant validity, concurrent validity with generic (Palliative care Outcome Scale) and condition specific measures (Multiple Sclerosis Impact Scale; Non-motor Symptoms Questionnaire; Parkinson’s Disease Questionnaire), responsiveness and minimally clinically important difference.

**Results:**

Of the 134 participants, MS patients had a mean Extended Disability Status Scale score 7.8 (SD = 1.0), patients with an IPD, MSA or PSP were in Hoehn & Yahr stage 3–5. The IPOS Neuro-S8 had high data quality (2% missing), mean score 8 (SD = 5; range 0–32), no ceiling effects, borderline floor effects, good internal consistency (Cronbach’s α = 0.7) and moderate test-retest reliability (intraclass coefficient = 0.6). The results supported a moderately correlated two-factor structure (Pearson’s r = 0.5). It was moderately correlated with generic and condition specific measures (Pearson’s r: 0.5–0.6). There was some evidence for discriminant validity in IPD, MSA and PSP (p = 0.020), and for good responsiveness and longitudinal construct validity.

**Conclusions:**

IPOS Neuro-S8 shows acceptable to promising psychometric properties in common forms of progressive LTNCs. Future work needs to confirm these findings with larger samples and its usefulness in wider disease groups.

## Introduction

Progressive long term neurological conditions (LTNC) are a group of irreversible and degenerative diseases of the nervous system that share some common characteristics, such as an increasing deterioration in neurological function over time, leading to increasing disability, cognitive impairment and dependence on others. As the disease progresses, complex physical, psychosocial and spiritual issues can arise which require palliative care and integrated care provisions from multiple agencies [1–3]; the primary care focus also moves from cure to comfort, and the patient perspectives become central [4]. Little research is conducted on how best to integrate and coordinate these care services, and on the effectiveness of different care models among this population. One major barrier may be the lack of appropriate patient centred palliative care outcome measures (PCOM).

A number of disease specific PCOMs are in existence for LTNCs, e.g. the Multiple Sclerosis Impact Scale (MSIS-29)[5], the Parkinson’s Disease Questionnaire (PDQ-39, PDQ-8) [6, 7], and Non-Motor Symptoms Questionnaire (NMSQuest) [8], but all of these measures fail to adequately address the progressive nature of the LTNC and the resulting complex needs including palliative care needs. For example, none of the existing measures feature pain, dyspnoea and other distressing symptoms which are frequently reported by patients and are recommended as triggers for palliative care referral [1, 2, 9, 10].

The 10-item palliative care outcome scale (core POS) is a widely used PCOM for palliative care patients. However, it was designed for general palliative care purposes and when applied to LTNCs it is not as sensitive as the measure primarily focusses on key symptoms that require palliative care input [10–12]. A brief PCOM for palliative care needs, comprising five typical symptoms (pain, nausea, vomiting, mouth problems and sleeping difficulty), was found to have satisfactory psychometric properties and was sensitive to change in patients with multiple sclerosis (MS) [11–13]. In an observational study in patients with idiopathic Parkinson’s disease (IPD), multiple system atrophy (MSA) and progressive supranuclear palsy (PSP), a 20-item measure covering more comprehensive aspects of palliative care needs including symptoms was used [14]. It appears to be a promising tool but has not yet been through formal psychometric evaluation. Based on the well validated core POS [15–17], several commonly used condition specific POS symptom versions [10–12], and clinical management guidelines for LTNCs [18, 19], we developed a new patient reported, integrated palliative care outcome measure that may be used to evaluate the outcome for people with progressive LTNC (IPOS Neuro).

A key component in the IPOS Neuro is the symptom burden, a concept that encompasses both the severity of the symptoms and the patient's perception of the impact of the symptoms [20]. This study provides a formal evaluation of the psychometric properties of the symptom burden specifically associated with eight core symptoms in the IPOS Neuro. For the convenience of reference, we named this subscale version the IPOS Neuro-S8. We examined five psychometric aspects: data quality, floor and ceiling effects, internal consistency and test-retest reliability, cross-sectional construct validity. We also provided a preliminary validation on responsiveness and longitudinal validity, and an initial estimate of the minimally clinically important difference (MCID).

## Data and Methods

### Ethical Approval

The Ethical approval for the MS trial was granted by the King’s College Hospital NHS Trust Local Research Ethics Committee (protocol number: 01-04-018); the IPD,MSA & PSP study was approved by both the Research Ethics Committee of the Institute of Psychiatry and King’s College Hospital NHS Foundation Trust (REC number: 05/Q0703/196). Written consent was given by all participants in both studies, through consent forms approved by the Research Ethics Committees.

### Setting and Design

Data was pooled from two studies: A) a randomised Phase II trial evaluating effectiveness of a short-term palliative care intervention in people severely affected by multiple sclerosis (MS)[11]; B) a longitudinal community based study in IPD, MSA and PSP [14]. Both studies were conducted in South East England (including London, Kent and Sussex) with the MS trial primarily focusing on patients living in South East London. Patients were recruited from a neurosciences Centre based at King’s College Hospital (KCH). This Centre is the second largest regional neuroscience centre in the UK and serves an estimated population of 3.5 million people.

### Eligibility Criteria

Patients with MS were included if their usual clinician deemed them to have one or more of unresolved symptoms, psychosocial concerns, end-of-life issues, progressive illness, or complex needs(i.e., palliative care needs); patients with a clinical diagnosis of IPD, MSA or PSP, who fell into Hoehn and Yahr (HY) stages 3–5 for IPD or equivalent motor disability for MSA and PSP [21].

In both studies, patients with urgent needs for symptom control, or support, or who were deteriorating rapidly or dying were excluded. Patients living in nursing homes were also excluded. The MS trial patients were randomly allocated to either the control or the intervention group, and the two groups were comparable for all factors. There was no clinically detectable change at 6 weeks on any clinically important variables [11].

### Measures

#### The 8 key symptom subscale of the Integrated Palliative care Outcome Scale for neurological conditions (IPOS Neuro-S8)

A generic, patient-centred palliative outcome measure of symptom burden for people with progressive LTNCs. It is based on a validated symptom burden measure in advanced multiple sclerosis—palliative care outcome scale, 5 symptoms (POS-MS-5)[11]. The POS-MS-5 assesses five key symptoms—pain, nausea, vomiting, mouth problems and sleeping difficulties over the past three days. To extend its use to the other neurological conditions, three further symptoms (breathlessness, spasms and constipation) prevalent in LTNCs are included to form the IPOS Neuro-S8 [18, 19, 22]. Each item is rated on a Likert scale from 0 (no problem) to 4 (overwhelming problem), with a total score range from 0 to 32 (S1 File). We propose to incorporate it into a 45-item integrated palliative care outcome measure (IPOS Neuro), with 37 items asking about symptom experience and the remaining items asking about information needs, practical concerns, anxieties of the patient and family, and their overall feeling of being at peace (S2 File).

#### The Core Palliative care Outcome Scale (Core POS)

A well validated and commonly used generic palliative care outcome measure [13, 15, 16]. It comprises of 10 items asking patients about their physical symptoms, information and practical needs, patient and family anxiety and well-being. The impact of the items on patients over the past three days is scored using a five-point Likert scale system, ranging from 0 (no problem) to 4 (overwhelming problems). The total score range is 0 to 40.

#### The Multiple Sclerosis Impact Scale (MSIS-29)

A disease-specific health related quality of life measure, with two subscales assessing physical and psychological impact of multiple sclerosis (MS)[5, 23]. It has a 20-item physical impact scale and a 9-item psychological impact scale. Each item on the MSIS has a five-point response option (1–5): “not at all,” “a little,” “moderately,” “quite a bit” and “extremely.” Scores on the physical impact scale can range from 20 to 100 and on the psychological impact scale from 9 to 45, with lower scores indicating little impact of MS and higher scores indicating greater impact. In this study, we used these two subscales.

#### The Parkinson’s disease Questionnaire (PDQ-8)

An 8-item self-completed, Parkinson’s disease specific questionnaire. Each item is rated: 0 (never) to 4 (always/cannot do) in the past one month. Items are: problems getting around in public, difficulty dressing self, felt depressed, embarrassed in public due to having disease, problems with close personal relationships, problems with concentration, unable to communicate with people properly, painful muscle cramps or spasms. The PDQ-8 Summary Index is expressed as a percentage of the sum of the items scores on the maximum possible scale score [6, 7].

#### The Non-Motor Symptoms Questionnaire (NMSQuest-30)

A 30-item self-administered, Parkinson’s disease specific questionnaire with three response categories (“yes”, “no” and “don’t know”) for each item. The items are divided into six domains: neuropsychiatric symptoms, autonomic disorders, disorders of smell, sleep disorders, sensory symptoms and other symptoms [8, 24]. Total NMSQuest scores range from 0 to 30 based on the total number of “yes” responses. Scores indicate how many different non-motor symptoms are present.

#### Other measures

The Extended Disability Status Scale (EDSS) is a single item 10-point clinician rated classification scheme used to assess physical disability for patients with MS [25]. The score range 0 (normal neurological exam) to 10 (death due to MS) with a step increase of 0.5. EDSS 1.0 to 4.5 refer to people with a high degree of ambulatory ability, 5.0 to 9.5 refer to the loss of ambulatory ability. The modified Hoehn & Yahr scale (HY) is a measure of the symptom progression for IPD, MSA and PSP, where stage 3, 4 and 5 respectively indicate “mild to moderate bilateral disease; some postural instability; physically independent”, “Severe disability; still able to walk or stand unassisted” and “Wheelchair bound or bedridden unless aided” [21]. The EuroQoL 5-Dimensions Questionnaire (EQ-5D) is a widely used generic instrument of health related quality of life [26]. It includes a descriptive part and a visual analogue scale for current health status (EQ-VAS, score range: 0 = the worst possible health status to 100 = the best possible health status). The descriptive part consists of five questions asking respondent’s current health in dimensions, i.e. mobility, self-care, usual activities, pain/discomfort and anxiety/depression. The rating on these five items is converted to a value (EQ-index) using the UK weighting schemes [27].

### Data Collection and Follow Up Schedule

Details of data collection and follow up schedule have been described elsewhere [11, 14, 28]. In brief, data were collected through face-to-face interviews in both studies. Socio-demographic information, clinical characteristics and the summary scores of patient reported measures were recorded at baseline. The patients in both studies were followed up at 12 weeks, and the patients with MS had a further data collection point at 6 weeks.

### Statistical Analysis

Total scores and item specific scores were described using mean and standard deviation (SD). Individual items were examined for their frequency distribution and expressed as a percentage. Floor and ceiling effects were measured by the number of respondents receiving the low (0–2) and high range (30–32) summary scores, or the minimum (0) and maximum (4) score on the individual items, respectively. The floor and ceiling effects were deemed present when more than 15% of patients recorded the extreme scores [29]. Data quality was assessed by the completeness of responses.

To evaluate the dimensions of the scale, exploratory factor analysis (EFA) and confirmatory factor analysis (CFA) were used. The EFA was conducted using the maximum likelihood method with promax rotation. The number of factors were determined by Spree plot and Kaiser’s criterion of an Eigen value>1. A factor loading greater than 0.30 was considered significant. The factorial structure of the scale was verified by CFA [30]. Several fit indices were selected to assess the CFA model fit: chi-square test statistics, root-mean-squared error of approximation (RMSEA), standardised root mean square residual (SRMR), and Comparative Fit Index (CFI) [31, 32]. RMSEA is a measure of the average of the residual variance and covariance; good models have RMSEA values that are at or less than 0.08. SRMR is a measure of the mean absolute value of the covariance residuals; values below 0.05 indicate good fit; CFI is an index that falls between 0 and 1, with values greater than 0.95 considered being indicators of good fitting models.

The internal consistency of the scale was calculated with Cronbach's alpha coefficient (minimum acceptable value for alpha was 0.7) [33]. Pearson’s correlation coefficient (r, 95%CI) was used to evaluate the concurrent and the convergent validity. The IPOS Neuro-S8 was anticipated to be more strongly correlated with physical domain than non-physical domain, e.g. in MS, higher in MSIS physical than MSIS psychological, and in IPD, MSA & PSP higher in NMSQuest than in PDQ-8. The interpretation of an absolute *r* value is as follows: ≤0.35—low or weak correlations, 0.36 to 0.67—modest or moderate correlations, and 0.68 to 1.0—strong or high correlations [34]. Discriminant validity was assessed using the known-groups validation method. Patients with higher level of disability were expected a priori to have a higher need for palliative care compared with patients with lower level of disability (EDSS<8 versus 8+ in MS, HY3 versus HY4&5 in IPD, MSA & PSP, respectively). The mean difference between groups was compared with the two sample t-test.

The test-retest reliability was explored with the intraclass correlation coefficient (ICC), using the baseline and six-week data from the MS trial only. The responsiveness of the IPOS Neuro-S8 was evaluated using the change scores from baseline to 12 week follow up. Two aspects were assessed and compared using independent t-test: responsiveness to intervention (MS trial), and to disease trajectory by diagnosis (IPD versus MSA & PSP). The responsiveness in MS sample was also analysed with effect size, interpreted using Cohen’s rules [35]. Longitudinal concurrent validity was tested using Pearson’s correlation coefficient r on the change scores between measures.

The minimal clinically important difference (MCID) was derived using the distribution based approach [36]. The MCID was estimated by the standard error of measurement (SEM), one half and one third of the standard deviation of the change score [37, 38]. Strictly speaking, SEM is a measure of the precision of the scale. As a precision measure, it has an arbitrary criterion (e.g. <1/2 or 1/3 SD) to be deemed as appropriate [39]. However, 1 SEM can be used as an approximation to the MID, along with other distribution-based methods for interpreting PROMs that produce dissimilar results [40].

All statistical tests were two-sided, P<0.05 was considered statistically significant. Data were analysed by SAS 9.4 (SAS Institute Inc, Cary, USA).

## Results

### Characteristics of the Study Participants

The pooled sample consists of 134 patients (Table 1). The average age of the study participants was 62 years old (SD = 12). Patients with MS were younger than patients with IPD, MSA or PSP (53 vs 66 years, p<0.001). Most patients with MS were women (69%), while gender was more balanced in IPD, MSA and PSP sample (55% vs 45%). 94% of patients with MS were in primary- or secondary-progressive stage (44% and 50%, mean EDSS score = 7.8(SD = 1.0)). Patients with MSA and PSP (71% in H&Y stage 4 & 5) were in more advanced disability stages than those with IPD (60% in H&Y stage 4 & 5). The disease duration was significantly shorter in MSA and PSP (4 years) than in MS (15.5 years) or IPD (12 years) (p<0.0001).

**Table 1:** Demographic and clinical characteristics in MS and IPD, MSA & PSP samples.

Characteristics	Value	All(n = 134)	MS(n = 52)	IPD, MSA & PSP			
				All(n = 82)	IPD(n = 50)	MSA(n = 17)	PSP(N = 15)
Age	Mean(SD)	61.6(12)	53.0(10)	66(9)	66.5(8)	67(8)	69(12)
	Median(min, max)	64(33,86)	52(33,75)	68(38, 86)	67(40,80)	68(51,81)	71(38,86)
Gender	Woman%	73(54%)	36(69%)	37(45%)	23(46%)	6(35%)	8(53%)
Diagnosis	Primary: secondary: other	-	23:26:3	NA			
Disease stage/progressiveness	Mean EDSS score(SD)	-	7.8(1.0)				
	Median EDSS (min-max)	-	8.0(5.5–9.5)				
	H&Y 3:4:5	-	-	29:37:34%	40:38:22%	12:41:47%	13:27:60%
Duration of illness since diagnosis	Mean(SD)	12.4(8.5)	17.2(9.6)	9.3(6.0)	11.9(5.8)	5.9(4.0)	4.6(2.7)
	Median(min, max)	12(0,54)	15.5(0,54)	8.0(1, 25)	12(1,25)	5(1,14)	4(1,11)
Employment	Retired	72(54%)	11(21%)	61(74%)	38(76%)	11(65%)	12(80%)
	In employment/self employed	7(5%)	1(2%)	6(7%)	6(12%)	9(0%)	0(0%)
	Unable to work(due to illness)	55(41%)	40(77%)	15(18%)	6(12%)	6(35%)	3(20%)
Ethnic group	White%	113(84%)	47(90%)	66(80%)	37(74%)	17(100%)	12(80%)
Living arrangement	Lives with carer	104(78%)	41(79%)	63(77%)	36(72%)	14(82%)	13(87%)
	Lives alone	23(17%)	9(17%)	14(17%)	10(20%)	2(12%)	2(13%)
	Other	7(5%)	2(4%)	5(6%)	4(8%)	1(6%)	0(0%)

Hoehn & Yahr stage refers to: 3 Mild to moderate bilateral disease; some postural instability; physically independent; 4 Severe disability; still able to walk or stand unassisted; 5 Wheelchair-bound or bedridden unless aided.

### Scale- and Item-Level Descriptive Statistics and Frequency Distribution

The mean total score of the IPOS Neuro-S8 was 7.8 (SD = 4.8); the score distribution appeared to be near normal with a kurtosis of -0.008 and a skewness of 0.54 (p value for Shapiro-Wilk test<0.001) (Table 2). All of the five response categories (0–4) were used in both conditions. Only a small proportion of patients scored the highest possible score (4) on the individual items. Other than nausea and vomiting, all items were roughly symmetrical around the score range 0–3. The mean total score of the IPOS Neuro-S8 was higher in IPD, MSA & PSP than in MS (9.0 vs 6.0, p<0.001); the average scores for the pain, shortness of breath, mouth problems and difficulty in sleep items followed a similar pattern and were significantly different between MS and IPD, MSA & PSP (p<0.05). No difference was found in spasms, nausea, vomiting and constipation scores by condition (p>0.09).

**Table 2:** Descriptive statistics of IPOS Neuro-S8 scores in the combined data and by condition.

Item	All (N = 134)							Multiple Sclerosis (N = 52)							IPD,MSA & PSP (N = 82)						
	Mean(SD)	%						Mean(SD)	%						Mean(SD)	%					
		0	1	2	3	4	missing		0	1	2	3	4	missing		0	1	2	3	4	missing
Total*	7.8(4.8)	14.9				0.0	1.5	6.0(4.7)	28.9				0.0	5.8	9.0(4.5)	6.1				0.0	0.0
Pain	1.8(1.2)	23.1	12.7	29.1	29.1	4.5	1.5	1.5(1.3)	30.8	21.2	19.2	19.2	5.8	3.8	2.0(1.1)	18.3	7.3	35.4	35.4	3.7	0.0
Spasms	1.4(1.2)	34.3	11.9	33.6	16.4	2.2	1.5	1.4(1.1)	26.9	21.2	32.7	11.5	3.8	3.8	1.4(1.2)	39.0	6.1	34.1	19.5	1.2	0.0
Shortness of breath	0.7(1.0)	57.5	19.4	14.9	6.0	0.7	1.5	0.3(0.7)	71.2	21.2	1.9	0.0	1.9	3.8	0.9(1.1)	48.8	18.3	23.2	9.8	0.0	0.0
Nausea	0.4(0.9)	73.9	11.9	7.5	3.7	1.5	1.5	0.4(0.9)	71.2	15.4	5.8	1.9	1.9	3.8	0.5(0.9)	75.6	9.8	8.5	4.9	1.2	0.0
Vomiting	0.2(0.6)	91.0	2.2	2.2	2.2	0.7	1.5	0.2(0.7)	86.5	3.8	3.8	0.0	1.9	3.8	0.1(0.6)	93.9	1.2	1.2	3.7	0.0	0.0
Constipation	1.2(1.2)	44.0	15.7	18.7	17.9	1.5	2.2	0.9(1.2)	50.0	17.3	13.5	11.5	1.9	5.8	1.3(1.2)	40.2	14.6	22.0	22.0	1.2	0.0
Spasms	1.4(1.2)	34.3	11.9	33.6	16.4	2.2	1.5	1.4(1.1)	26.9	21.2	32.7	11.5	3.8	3.8	1.4(1.2)	39.0	6.1	34.1	19.5	1.2	0.0
Difficulty in sleep	1.1(1.3)	47.8	11.9	20.1	14.9	3.0	2.2	0.8(1.3)	59.6	9.6	11.5	7.7	5.8	5.8	1.3(1.2)	40.2	13.4	25.6	19.5	1.2	0.0
Mouth problems	1.1(1.2)	45.5	17.9	18.7	13.4	3.0	1.5	0.5(1.0)	73.1	5.8	13.5	1.9	1.9	3.8	1.5(1.2)	28.0	25.6	22.0	20.7	3.7	0.0

*0–2 for the minimum, and 30–32 for the maximum in case of total score; 0 for the minimum and 4 for the maximum in item-specific score. Floor or ceiling effects are considered to be present if more than 15% of respondents fell in the extreme score categories[29].

### Missing Data, Floor and Ceiling Effects

The rate of missing data for the IPOS Neuro-S8 was low, with only 5.8% of total scores missing in the MS sample and no missing for IPD, MSA & PSP patients (Table 2). The items missing in the MS data was in a small range (3.8–5.8%). The missing pattern appeared to be scale missing and was not related to individual items or observed factors. There was evidence for a borderline floor effect, with 14.9% of the patients scoring 0–2; the floor effect was evident in patients with MS (28.9%) but not for IPD, MSA & PSP patients (6.1%). There was no ceiling effect for the total score in the combined data or for both diagnostic groups. The floor effect was present for all items, with 23 to 91% of the patients receiving the lowest possible score (0).

### Factor Structure

The EFA indicated a two-factor structure (P = 0.67, *X*^*2*^ *=* 10.2, df = 13), with factor 1 loaded with five items and factor two with three items (Table 3). All fit indices in the CFA met acceptable level model fit. The two factors were moderately correlated (Pearson’s r = 0.48), suggesting two associated but distinct constructs. The factor loadings on spasms (0.31 for factor 1) and on constipation (0.28 for factor 2) were weak.

**Table 3:** Standardized Factor Loadings (Standard Error, SE), factor correlation (SE) and the fit indices in the confirmatory factor analysis of the IPOS Neuro-S8.

Item	Factor1(5 items)	Factor2(3 items)
Pain	0.61(0.09)	
Shortness of breath	0.43(0.10)	
*Nausea*		0.99(0.10)
*Vomiting*		0.64(0.08)
*Constipation*		0.28(0.09)
Spasms	0.31(0.10)	
Difficulty in sleep	0.49(0.09)	
Mouth problems	0.49(0.09)	
Correlation of the two factors	0.48(0.11)	
Fit statistics	P = 0.19(Chi-square = 24.2, DF = 19)	
	Standardised RMR(SRMR) = 0.057	
	RMSEA = 0.046	
	Comparative fit index = 0.9634	

### Internal Consistency and Test-Retest Reliability

The Cronbach’s alpha for the combined sample was 0.66; it was higher in MS (0.70) than in patients with IPD, MSA or PSP (0.60). Given that the IPOS Neuro-S8 had only eight items with two moderately correlated factors, we interpreted the internal consistency of the scale as acceptable. The test-retest analysis revealed the agreement between the baseline and the week 6 assessments was fair, with an ICC value of 0.58 (95%CI: 0.28–0.83). The baseline total scores were not significantly different from those of week 6 (p for paired t-test = 0.80).

### Construct Validity

The IPOS Neuro-S8 was moderately correlated with the core POS (Pearson r 0.50, 95%CI: 0.36–0.62) (Table 4). The correlation between IPOS Neuro-S8 and core POS were similar (r = 0.58) in the two diagnostic groups. The correlation with existing disease specific measures (MSIS-Physical/ Psychological, PDQ, NMSQuest) was also moderate, ranging from 0.46 to 0.59 but statistically significant (p<0.05). The IPOS Neuro-S8 was negatively correlated with both EQ-5D and health status (-0.39 and -0.25), and positively correlated with the HADS-14 (0.29).

**Table 4:** Cross-sectional and longitudinal reliability and validity of the IPOS Neuro-S8.

Psychometric properties	Measure	Time scale	All (N = 134)	MS (N = 52)	IPD,MSA &PSP (N = 82)
Cross-sectional					
Internal consistency (Cronbach alpha)	Full scale	T0	0.66	0.70	0.60
Construct validity					
Concurrent (Pearson’s r, 95%CI)	Core POS	T0	0.50(0.36 to 0.62)	0.58(0.36 to 0.74)	0.58(0.41 to 0.71)
	MSIS physical	T0	-	0.59(0.36 to 0.75)	-
	MSIS psychological	T0	-	0.46(0.20 to 0.66)	-
	NMSQuest	T0	-	-	0.58(0.43 to 0.71)
	PDQ-8	T0	-	-	0.48(0.29 to 0.63)
Discriminative or known group (Mean(SD))	EDSS (<8 vs 8+)	T0	-	4.8(2.1) vs 6.9(5.6)^1^	-
	H&Y 3 vs 4&5	T0		-	7.2(4.1) vs 9.7(4.5)^2^
Convergent (Pearson’s r, 95%CI)	Factor 1 vs total score	T0	0.92(0.88 to 0.94)	0.91(0.85 to 0.95)	0.91(0.86 to 0.94)
	Factor 2 vs total score	T0	0.71(0.62 to 0.79)	0.73(0.57 to 0.84)	0.71(0.58 to 0.80)
	Factor 1 vs Factor 2 score	T0	0.37(0.22 to 0.51)	0.40(0.14 to 0.61)	0.35(0.14 to 0.52)
Longitudinal					
Test-retest reliability	ICC (95%CI)	T0 vs wk6	-	0.62(0.36 to 0.82)	-
Construct validity					
Concurrent (Pearson’s r, 95%CI)	Core POS	T0 to wk12	0.30(0.12 to 0.45)	0.16(-0.13 to 0.42)	0.56(0.38 to 0.70)
	MSIS physical	T0 to wk12	-	0.41(0.12 to 0.63)	-
	MSIS psychological	T0 to wk12	-	0.05(-0.25 to 0.33)	-
	NMSQuest	T0 to wk12	-	-	0.18(-0.06 to 0.39)
	PDQ-8	T0 to wk12	-	-	0.27(0.03 to 0.47)
Responsiveness(change score)	Mean (SD, 95%CI)	T0 to wk12	-0.42(3.4, -1.02 to 0.19)	-0.15(4.1, -1.33 to 1.04)	-0.60(2.8, -1.26 to 0.06)
To intervention: Intervention vs standard care			-	-1.31(4.3, -3.04 to 0.43) vs 1.23(3.4, -0.28 to 2.74)^3^	-
To disease trajectory by type of diagnosis (IPD vs MSA & PSP)			-		-0.45(2.9, -1.29 to 0.40) vs -0.88(2.7, -1.99 to 0.23)^4^
Minimally important difference(MID)*: distribution based approach	SEM	T0 to wk12	3.1	2.9	2.9
	1/2 SD	T0 to wk12	1.7	2.1	1.4
	1/3 SD	T0 to wk12	1.1	1.4	0.9

1: p for two sample t-test = 0.08

2: p for two sample t-test = 0.020

*Standard error of measurement (SEM) = SD_baseline_ × sqrt (1-ICC). The MID measures for MS group was based on the patients in the intervention group only.

3: mean (sd, 95%CI) for intervention and standard care group, respectively. The difference was significant (p = 0.030).

4: mean (sd, 95%CI) for IPD and the MSA&PSP combined, respectively. The difference was not statistically significant (p = 0.54).

The mean scores were different according to the EDSS score in MS sample (4.8 for EDSS <8 versus 6.9 for EDSS 8+), the difference did not reach the significance level (p = 0.08). In the IPD, MSA & PSP group, the H&Y staging system distinguished patients well, with higher scores corresponding to more severe symptoms (7.2 H&Y stage 3 vs 9.7 H&Y stage 4 & 5), and the difference was statistically significant (p = 0.020).

The two factors identified in the CFA were both strongly correlated with the total score (Pearson’s r = 0.91 for Factor 1 and 0.71 for Factor 2); but were only weakly related to each other (Pearson’s r = 0.35). The association pattern was similar in both conditions, providing further proof that the two factors reflect a different but related symptom profile.

### Responsiveness, MID and Longitudinal Construct Validity

The IPOS Neuro-S8 was responsive to change. This was more evident for patients with MS when comparing the patients in the intervention arm to those in the control arm. The t-test result was statistically significant (p = 0.030, t = 2.23, df = 46). The score change in IPD and the combined MSA & PSP was in the same direction (mean change -0.45 and -0.88), but none of the changes reached significance level and the changes between groups were also not significant (p = 0.54, t = 0.62, df = 70). The effect size (ES) of the short-term palliative care intervention in MS patients was small (<0.5). The MID was 3.1 as measured by SEM, greater than half and a third of SD (1.7 and 1.1).

Longitudinally, the IPOS Neuro-S8 showed reasonable correlation with the core POS and with the disease specific measures. However, few of them reached statistical significance due to the sample size reduction caused by attrition.

## Discussion

This study provided initial evidence on the cross sectional and longitudinal reliability and validity of the IPOS Neuro-S8, a new PCOM for people with progressive LTNCs. The IPOS Neuro-S8 showed acceptable to promising psychometric properties for use across neurological conditions, including advanced MS, late stage progressive IPD, PSP and MSA. While most studies involving severely ill people often suffer from small numbers [41, 42], this evaluation was based on a relatively large sample (N = 134) therefore making it statistically more robust. The reliability of the IPOS Neuro-S8 appears adequate based on a Cronbach’s alpha value of 0.66 with only 8 items, and supported by the two factor solution and their moderate correlation (Pearson’s r = 0.48) [43, 44]. The IPOS Neuro-S8 is correlated reasonably well with the non-condition specific core POS. The core POS, as a multidimensional measure, only two out of its ten items are questions about pain and one other physical symptom. Therefore, a high correlation between IPOS Neuro-S8 and core POS is not anticipated.

The correlation between IPOS Neuro-S8 and the diagnosis specific scales (MSIS-29, NMSQuest-30 and PDQ-8) was as expected (Pearson’s r range: 0.46–0.59), e.g. higher in physical and lower in psychological aspects. One reason for the moderate association may be that none of these measures were designed specifically for palliative care needs or for people severely affected by their illness; thus, intrinsically these measures may not capture the same needs as those captured by the IPOS Neuro-S8 [5, 7, 8, 23]. The time frames used in the disease specific measures may be another reason. For example, NMQuest-30 and PDQ-8 ask symptoms experienced over the past month (or 4 weeks) and the MSIS is based on the past two weeks. In contrast, the IPOS Neuro-S8 asks about the past three days, aiming to capture the immediate care needs. For patients with neurological conditions who require palliative care input, the disease trajectory is more unpredictable and more frequent assessments are recommended as best practice [9].

Our results support the continued development and validation of the IPOS Neuro-S8. If proved by further validation studies, it can be a useful outcome measure in research settings, particularly for patients with progressive LTNCs, as it is brief and shows promising psychometric properties. It may also facilitate the clinical use of the IPOS Neuro-S8 as a screening measure in non-palliative care settings, to increase the opportunities of identifying patients with palliative care needs and trigger the referral process. In palliative care clinical practice, it may be used to monitor care intervention outcomes and disease progression. At the organisation level, it can be a simple, standardised auditing tool. It also makes patient’s self-monitoring possible.

Several limitations are noted in our study. We could not test face validity and content validity in this study, but all eight items of the IPOS Neuro-S8 were important for patients and relevant in clinical practices [9, 18]. Although having been able to assess a range of psychometric properties, this evaluation was based on two existing datasets using very different study designs–a longitudinal observational study and a randomised clinical trial. Previous research revealed that eligibility criteria for patient selection in clinical trials can be very restrictive, therefore these patient samples may not be as representative as those from observational studies [45]. Some very “severe” patients were excluded as well–less than 5% got the highest possible score on the individual items; we did not have data for other prevalent neurological conditions that may have a higher need for palliative care, e.g. motor neuron diseases. As a consequence of the high attrition rate and the loss of statistical power, the longitudinal psychometric evaluation should be treated as preliminary. Furthermore, the psychometric testing in this study was primarily based on classical test theory; it may be of interest to compare how the results differ from those derived from modern measurement theories.

## Conclusions

In conclusion, our results demonstrated the acceptable to promising psychometric properties of the IPOS Neuro-S8 in two common long term neurological conditions. The IPOS Neuro-S8 showed good data quality, borderline floor effects and no ceiling effects, good internal consistency and moderate test-retest reliability. A moderately correlated two-factor structure was confirmed. The IPOS Neuro-S8 was moderately correlated with both the generic core POS and the condition specific measures (MSIS-29, NMSQuest-30, PDQ-8). There was evidence for discriminant validity in IPD, MSA and PSP (by disease progression). Preliminary evidence suggested good responsiveness and longitudinal construct validity but needs further research. Future work needs to confirm these findings with larger samples and its usefulness in wider LTNC disease groups.

## Supporting Information

S1 File The 8 key symptoms of the Integrated Palliative Outcome Scale for patients with long term neurological condition (IPOS Neuro-S8). (DOCX)

S2 File The Integrated Palliative Outcome Scale for patients with long term neurological condition (IPOS Neuro). (DOCX)
