# Supplementary material for: Psychometric Properties of a Generic, Patient-Centred Palliative Care Outcome Measure of Symptom Burden for People with Progressive Long Term Neurological Conditions
Source: PLoS One. 2016 Oct 25;11(10):e0165379. doi: 10.1371/journal.pone.0165379 (PMC5079599; doi:10.1371/journal.pone.0165379)
Supplement: S1 File — (DOCX) [file pone.0165379.s001.docx]

S1 File. The 8 key symptoms of the Integrated Palliative Outcome Scale for patients with Long term neurological condition (IPOS Neuro-S8)

**IPOS Neuro-S8 Patient Version**

**Below is a list of symptoms, which you may or may not have experienced. For each symptom, please tick one box that best describes how it has affected you over the past 3 days.**

|  | **Not at all** | **Slightly** | **Moderately** | **Severely** | **Over-whelmingly** |
| --- | --- | --- | --- | --- | --- |
| **Pain** | 0□ | 1□ | 2□ | 3□ | 4□ |
| **Spasms** | 0□ | 1□ | 2□ | 3□ | 4□ |
| **Shortness of breath** | 0□ | 1□ | 2□ | 3□ | 4□ |
| **Nausea** | 0□ | 1□ | 2□ | 3□ | 4□ |
| **Vomiting** | 0□ | 1□ | 2□ | 3□ | 4□ |
| **Mouth Problems** | 0□ | 1□ | 2□ | 3□ | 4□ |
| **Difficulty in sleeping** | 0□ | 1□ | 2□ | 3□ | 4□ |
| **Constipation** | 0□ | 1□ | 2□ | 3□ | 4□ |
