# Supplementary material for: Psychometric Properties of a Generic, Patient-Centred Palliative Care Outcome Measure of Symptom Burden for People with Progressive Long Term Neurological Conditions
Source: PLoS One. 2016 Oct 25;11(10):e0165379. doi: 10.1371/journal.pone.0165379 (PMC5079599; doi:10.1371/journal.pone.0165379)
Supplement: S2 File — (DOCX) [file pone.0165379.s002.docx]

S2 File. The Integrated Palliative Outcome Scale for patients with long term neurological condition (IPOS Neuro)

**IPOS Neuro Patient Version**

**Q1. What have been your main problems or concerns over the past 3 days?**

1.....................................................................................................................................

2.....................................................................................................................................

3. ...................................................................................................................................

**Q2. Below is a list of symptoms, which you may or may not have experienced. For each symptom, please tick one box that best describes how it has affected you over the past 3 days.**

|  | **Not at all** | **Slightly** | **Moderately** | **Severely** | **Over-whelmingly** |
| --- | --- | --- | --- | --- | --- |
| **Pain** | 0□ | 1□ | 2□ | 3□ | 4□ |
| **Shortness of breath** | 0□ | 1□ | 2□ | 3□ | 4□ |
| **Nausea (feeling like you are going to be sick)** | 0□ | 1□ | 2□ | 3□ | 4□ |
| **Vomiting (being sick)** | 0□ | 1□ | 2□ | 3□ | 4□ |
| **Poor appetite** | 0□ | 1□ | 2□ | 3□ | 4□ |
| **Constipation** | 0□ | 1□ | 2□ | 3□ | 4□ |
| **Sore or dry mouth** | 0□ | 1□ | 2□ | 3□ | 4□ |
| **Drowsiness** | 0□ | 1□ | 2□ | 3□ | 4□ |
| **Poor mobility** | 0□ | 1□ | 2□ | 3□ | 4□ |
| **Spasms** | 0□ | 1□ | 2□ | 3□ | 4□ |
| **Fatigue or lack of energy** | 0□ | 1□ | 2□ | 3□ | 4□ |
| **Problems swallowing** | 0□ | 1□ | 2□ | 3□ | 4□ |
| **Feeling sleepy** | 0□ | 1□ | 2□ | 3□ | 4□ |
| **Difficulty in sleeping** | 0□ | 1□ | 2□ | 3□ | 4□ |
| **Difficulty with bowel control** | 0□ | 1□ | 2□ | 3□ | 4□ |
| **Difficulty controlling urine** | 0□ | 1□ | 2□ | 3□ | 4□ |
| **Pressure Sores** | 0□ | 1□ | 2□ | 3□ | 4□ |
| **Problems using your arms** | 0□ | 1□ | 2□ | 3□ | 4□ |
|  | **Not at all** | **Slightly** | **Moderately** | **Severely** | **Over-whelmingly** |
| **Problems using your legs** | 0□ | 1□ | 2□ | 3□ | 4□ |
| **Difficulty communicating** | 0□ | 1□ | 2□ | 3□ | 4□ |
| **Dribbling of Saliva** | 0□ | 1□ | 2□ | 3□ | 4□ |
| **Falls** | 0□ | 1□ | 2□ | 3□ | 4□ |
| **Hallucinations** | 0□ | 1□ | 2□ | 3□ | 4□ |
| **Mouth Problems** | 0□ | 1□ | 2□ | 3□ | 4□ |
| **Loss or change in your ability to taste or smell** | 0□ | 1□ | 2□ | 3□ | 4□ |
| **Unexplained change in weight (not due to change in diet).** | 0□ | 1□ | 2□ | 3□ | 4□ |
| **Problems remembering things that have happened recently or forgetting to do things.** | 0□ | 1□ | 2□ | 3□ | 4□ |
| **Loss of interest in what is happening around you or in doing things.** | 0□ | 1□ | 2□ | 3□ | 4□ |
| **Difficulty concentrating or staying focused.** | 0□ | 1□ | 2□ | 3□ | 4□ |
| **Feeling less interested in sex or more interested in sex.** | 0□ | 1□ | 2□ | 3□ | 4□ |
| **Finding it difficult to have sex when you try.** | 0□ | 1□ | 2□ | 3□ | 4□ |
| **Feeling light-headed, dizzy or weak standing from sitting or lying.** | 0□ | 1□ | 2□ | 3□ | 4□ |
| **Excessive sweating** | 0□ | 1□ | 2□ | 3□ | 4□ |
| **Double vision** | 0□ | 1□ | 2□ | 3□ | 4□ |

**Please list any other symptoms not mentioned above, and tick one box to show how they have affected you over the past 3 days.**

|  | **Not at all** | **Slightly** | **Moderately** | **Severely** | **Over-whelmingly** |
| --- | --- | --- | --- | --- | --- |
| **1.** | 0□ | 1□ | 2□ | 3□ | 4□ |
| **2.** | 0□ | 1□ | 2□ | 3□ | 4□ |
| **3.** | 0□ | 1□ | 2□ | 3□ | 4□ |

**Over the past 3 days:**

|  | **Not at all** | **Occasionally** | **Sometimes** | **Most of the time** | **Always** |
| --- | --- | --- | --- | --- | --- |
| **Q3**. **Have you been feeling anxious or worried about your illness or treatment?** | 0□ | 1□ | 2□ | 3□ | 4□ |
| **Q4**. **Have any of your family or friends been anxious or worried about you?** | 0□ | 1□ | 2□ | 3□ | 4□ |
| **Q5**. **Have you been feeling depressed?** | 0□ | 1□ | 2□ | 3□ | 4□ |
|  | | | | | |
|  | **Always** | **Most of the time** | **Sometimes** | **Occasionally** | **Not at all** |
| **Q6**. **Have you felt at peace?** | 0□ | 1□ | 2□ | 3□ | 4□ |
| **Q7**. **Have you been able to share how you are feeling with your family or friends as much as you wanted?** | 0□ | 1□ | 2□ | 3□ | 4□ |
| **Q8**. **Have you had as much information as you wanted?** | 0□ | 1□ | 2□ | 3□ | 4□ |

**Over the past 3 days:**

|  | **Problems addressed/ No problems** | **Problems mostly addressed** | **Problems partly addressed** | **Problems hardly addressed** | **Problems not addressed** |
| --- | --- | --- | --- | --- | --- |
| **Q9**. **Have any practical problems resulting from your illness been addressed? (such as financial or personal)** | 0□ | 1□ | 2□ | 3□ | 4□ |

|  | **None at all** | **Up to half a day wasted** | **More than half a day wasted** |
| --- | --- | --- | --- |
| **Q10. How much time do you feel has been wasted on appointments relating to your healthcare, e.g. waiting around for transport or repeating tests?** | 0□ | 2□ | 4□ |
|  | | | |
|  | **On my own** | **With help from a friend or relative** | **With help from a member of staff** |
| **Q11. How did you complete this questionnaire?** | 1□ | 2□ | 3□ |
